# Phylogenetic Relationships and Next-Generation Barcodes in the Genus *Torreya* Reveal a High Proportion of Misidentified Cultivated Plants

**DOI:** 10.3390/ijms241713216

**Published:** 2023-08-25

**Authors:** Zhi-Qiong Mo, Jie Wang, Michael Möller, Jun-Bo Yang, Lian-Ming Gao

**Affiliations:** 1 CAS Key Laboratory for Plant Diversity and Biogeography of East Asia, Kunming Institute of Botany, Chinese Academy of Sciences, Kunming 650201, China; 2 University of Chinese Academy of Sciences, Beijing 100049, China; 3 Germplasm Bank of Wild Species, Kunming Institute of Botany, Chinese Academy of Sciences, Kunming 650201, China; 4 Royal Botanic Garden Edinburgh, Edinburgh EH3 5LR, UK; 5 Lijiang Forest Biodiversity National Observation and Research Station, Kunming Institute of Botany, Chinese Academy of Sciences, Lijiang 674100, China

**Keywords:** *Torreya*, genome skimming, species identification, phylogeny

## Abstract

Accurate species identification is key to conservation and phylogenetic inference. Living plant collections from botanical gardens/arboretum are important resources for the purpose of scientific research, but the proportion of cultivated plant misidentification are un-tested using DNA barcodes. Here, we assembled the next-generation barcode (complete plastid genome and complete nrDNA cistron) and mitochondrial genes from genome skimming data of *Torreya* species with multiple accessions for each species to test the species discrimination and the misidentification proportion of cultivated plants used in *Torreya* studies. A total of 38 accessions were included for analyses, representing all nine recognized species of genus *Torreya*. The plastid phylogeny showed that all 21 wild samples formed species-specific clades, except *T. jiulongshanensis*. Disregarding this putative hybrid, seven recognized species sampled here were successfully discriminated by the plastid genome. Only the *T. nucifera* accessions grouped into two grades. The species identification rate of the nrDNA cistron was 62.5%. The Skmer analysis based on nuclear reads from genome skims showed promise for species identification with seven species discriminated. The proportion of misidentified cultivated plants from arboreta/botanical gardens was relatively high with four accessions (23.5%) representing three species. Interspecific relationships within *Torreya* were fully resolved with maximum support by plastomes, where *Torreya jackii* was on the earliest diverging branch, though sister to *T. grandis* in the nrDNA cistron tree, suggesting that this is likely a hybrid species between *T*. *grandis* and an extinct *Torreya* ancestor lineage. The findings here provide quantitative insights into the usage of cultivated samples for phylogenetic study.

## 1. Introduction

Accurate species identification is key to conservation and biological research, such as the inference of phylogenetic relationships and unravelling the biogeographic history of taxa. The availability and affordability of next-generation sequencing (NGS) technologies have greatly expanded the use of genomic data. For instance, the next-generation barcodes (the completed plastome and the nuclear ribosomal DNA cistron (nrDNA), consisting of the 18S small-subunit (SSU) of the ribosomal RNA (rRNA) gene, the internal transcribed spacer 1 (ITS1), the 5.8S rRNA gene, the internal transcribed spacer 2 (ITS2), and the 26S large-subunit (LSU) rRNA gene) easily assembled from genome skimming data, have shown a great promise for reliably distinguishing closely related species [1,2,3,4,5]. In addition, the plastid genome contains a large number of evolutionarily informative variations, which are widely used for phylogenetic reconstruction at deep to shallow levels in land plants [6,7,8,9,10]. Crucially, the inclusion of multiple individuals from different populations across the entire distribution range of all species is needed to ensure the accurate species delimitation and correct inference and interpretation subsequently of their phylogenetic relationships [11,12]. However, very few studies hitherto have used multiple individuals per species with genomic data or included all species within a given genus.

Plant material for most endangered, rare and narrowly distributed species are difficult to gather in the field, whereas botanical gardens and arboretums typically hold a wide diversity of well-documented living plant collections, which are important resources for the purpose of scientific research, conservation, display and education [13]. However, the morphological characters of plants growing in ex situ conditions may change, which may lead to misidentification if the plants lack complete or clear origin information. In addition, mislabeling and misidentification before and after introduction into botanical gardens may also be a major cause of species misidentification. Misidentification of species collected from botanical gardens were reported in recent studies [11,13,14], which caused the incorrect inference of phylogenetic relationships and biogeographic history inferences. For instance, Le et al. [13] found that 110 out of 669 palm samples (16.4%) collected from botanical gardens were misidentified, which can also be a problem for reintroduction efforts. De Luca et al. [15] confirmed some *Erythrina* plants introduced or now cultivated in the ancient gardens of Naples were misidentified. Therefore, misidentification may spread misleading knowledge and result in the failure of species conservation, whereas verifying the identification of cultivated plant samples using DNA barcodes possibly can avoid misidentification leading to mistakes in biological studies or in conservation-linked reintroduction programs [13].

*Torreya* is a small coniferous genus in the family Taxaceae with an East Asia (EA)–North America (NA) disjunctive distribution [16,17], comprising six to eight species depending on the taxonomy classification followed [18,19,20,21]. Two species, *Torreya taxifolia* Arn. and *T. californica* Torr., occur in West and Southeast North America, respectively. *Torreya taxifolia* is restricted to a few ravines along the Appalachicola River in northern Florida and southern Georgia, and it is currently listed as critically endangered on The IUCN Red List of Threatened Species [22]. *Torreya californica* is endemic to the coastal ranges and Sierra Nevada of California and listed as vulnerable [16,23]. The other species are distributed in East Asia, they usually occur in the understory of broad-leaved or mixed broad-leaved-conifer forest [18]. *Torreya nucifera* Siebold and Zucc. is confined to Japan and Korea, and the remaining taxa (*T. grandis* Fortune ex Lindl., *T. jackii* Chun, *T. fargesii* Franch., *T. yunnanensis* W.C. Cheng and L.K. Fu, and *T. parvifolia* T.P. Yi, Lin Yang and T.L. Long) are native to China [18,20,21,24]. Of these, *T. fargesii* is listed as vulnerable [25], and *T. jackii* [26] and *T. yunnanensis* are listed as endangered species [27]. The taxonomy of *Torreya* is primarily based on morphological characters and geographical distribution of individual taxa, and the species delimitation is controversial [18,20,24]. For instance, Farjon [18] recognized six species and two varieties in *Torreya*. In this classification, *T. parvifolia*, confined to SW Sichuan province, was merged into *T. grandis* occurring in East China, and *T. yunnanensis*, native to Northwest Yunnan province, was treated as a variety of *T. fargesii*, distributed in Central China. On the other hand, Yang et al. [20] treated the two taxa, *T. parvifolia* and *T. yunnanensis*, as distinct species. Also, *T. grandis* var. *jiulongshanensis* Zhi Y. Li, Z.C. Tang and N. Kang, endemic to South Zhejiang of East China, was recently treated as an independent species [28]. This species was later shown to be a likely hybrid origin between two sympatric species, *T. jackii* and *T. grandis*, with the latter being the paternal parent [29]. Therefore, currently, nine species are recognized in *Torreya*, though more evidence to confirm their genetic species boundaries are needed, such as the entire plastome as next-generation barcode, which has shown great ability for distinguishing species in Taxaceae [11,12].

The monophyly of *Torreya* is supported by both morphology and molecular data [30,31]. However, published phylogenetic species relationships within the genus are conflicting, sometimes even when based on the same genomic markers [14,16,29,31,32,33]. For instance, the phylogenetic analysis of seven species based on nuclear RAD-seq genomic data resolved two monophyletic clades, the NA species clade and the EA species clade, the latter being: (*T. fargesii + T. yunnanense*) (*T. jackii* (*T. grandis* + *T. nucifera*)) with highly supported branches [14]. A plastome phylogeny showed a discordant phylogenetic position of *T. jackii*, which shifted to a position as sister to the rest of all other species [14]. However, also based on the complete plastome, the interspecific relationships differed from the above study in which *T. jackii* was also recovered as the earliest divergent species, but followed by *T. taxifolia*, rather than a sister clade consisting of NA species clade and other EA species clade [30,31,34,35]; other relationships saw *T. californica* as sister to *T. grandis* [30,31,34,35], and *T. yunnanense* was resolved as sister to a clade of *T*. *parvifolia* (*T. fargesii* + *T. nucifera*), and these two clades form a sister relationship [30,34]. These species formed a clade but had inconsistent relationships in Miao et al. [35] and Ji et al. [31]. Here, *T*. *nucifera* was sister to *T*. *californica* + *T*. *grandis*, while *T*. *yunnanense* clustered with *T*. *parvifolia* to form a sister relationship with *T*. *fargesii* + *T*. *nucifera* in Miao et al. [35]. *Torreya nucifera* was sister to a clade of *T. yunnanensis* (*T*. *californica* + *T*. *grandis*), and then formed a sister relationship with *T. parvifolia* + *T. fargesii* in Ji et al. [31].

As several species in *Torreya* are listed as endangered species with small distribution ranges, the plant material of some species used for previous molecular phylogenies came from cultivated plants in botanical gardens/arboretums, and only a single accession for each species was sampled in previous studies [30,31,33,34,35]. The misidentification of this cultivated plant material may have led to the discordances between the previous studies. For instance, the samples of *T*. *californica* and *T*. *nucifera* (GenBank ID: MK249062 and MK249060, respectively), collected from botanical gardens and used in Zhang et al. [30], were suspected to be misidentified based on their phylogenetic placement and/or examination of their photographs [14]. In any case, thus far, the proportion of cultivated plant accessions of *Torreya* misidentified in previous studies is unknown.

The phylogenetic discordance between nuclear and organelle, and between organelles (plastome and mitochondrion) may result from hybridization and/or introgression, and plastome capture events, which are due to the different inheritance patterns of the genomes [36]. The plastid genome is paternal inheritance in some Taxaceae [37,38], while the inheritance of the mitochondrial genome can be paternal or maternal in conifers and can include recombination [37,39], though it is partly maternal in Taxaceae [40], or seemingly maternal for both mitochondria and plastids in *Torreya* [41]. Comparing the concordance and/or discordance of nuclear and organelle phylogenies may aid to explore the evolutionary history and the inheritance model of the organelle within *Torreya*.

In this study, we acquired the next-generation barcode (complete plastid genome and nrDNA cistron) and mitochondrial genes for all *Torreya* species currently accepted [18,20,28], except for the recently described species *T*. *dapanshanica* X.F. Jin, Y.F. Lu and Zi L. Chen [42]. We included multiple individuals per species. We also added all available published complete plastomes, nrDNA cistrons and mitochondrial genes from the same individuals for analysis. A Skmer [43] analysis, which can use the entire nuclear reads generated in a genome skim as the identifier of a species [44] rather than just the highly repetitive nrDNA sequences, was also used for *Torreya* species identification. Explicitly, we aimed to: (1) evaluate the efficiency of the next-generation barcode and nuclear genome information obtained by Skmer for species delineation of species within *Torreya*; (2) determine the proportion of misidentification of cultivated plants from botanical gardens; (3) infer the phylogenetic species’ relationships within *Torreya*.

## 2. Results

### 2.1. Plastomes, Mitochondrial Genes and nrDNA Cistrons of Torreya

Complete circular plastid genomes of the 19 newly sequenced *Torreya* individuals were assembled with high quality, with an average sequencing coverage of 223 to 1144 × (Table 1). The plastomes of all *Torreya* accessions had a length ranging from 136,693 bp to 137,412 bp. Their average GC content was similar (35.4–35.5%; Table 1). The plastomes consisted of 118 unique genes, comprising 83 protein-coding genes, 31 tRNA genes and four rRNA genes. Of these, three genes, *trnI-CAU*, *trnN-GUU* and *trnQ-UUG*, each had two copies. Similar to the published plastomes of *Torreya* (e.g., *T. parvifolia* NC_043866, *T. taxifolia* MK249063 and *T. jackii* KX902234), an inverted repeat region was not found in the newly assembled plastomes.

A total of 26 mitochondrial genes and the complete nrDNA cistron of 26 *Torreya* samples and two species of *Amentotaxus* were assembled and extracted. Of these, the mitochondrial genes of one *Torryeya* sample (SRR10768423) had many missing data and was removed from the mitochondrial phylogenetic analyses. 

The length of the three unfiltered genomic sequence matrices, CP, MT and nrDNA, was 145,775 bp, 21,493 bp, and 6137 bp, respectively. For the samples of *Torreya*, Matrix CP had 2561 variable sites (1.82%) and 2063 parsimony informative (PI) sites (1.47%). The nrDNA cistron matrix had 52 variable (0.85%) and 44 PI (0.72%) sites, among which ITS1 accounted for the largest proportion (39 variable and 34 PI sites), followed by ITS2 (6 sites for both), 28S (5 variable and 3 PI sites), and 5.8S (2 variable and 1 PI sites), while 18S showed no variation. Matrix MT had the lowest proportions of both variable (0.07%; 14 total) and PI (0.03%; 7 total) sites among the three matrices. The proportion of variable and PI sites in the filtered matrices changed only slightly, and the filtered Matrix MT still contained the lowest values (Table 2).

### 2.2. Phylogenetic Analyses—Species Delineation and Sample Identities

**Figure 1:**
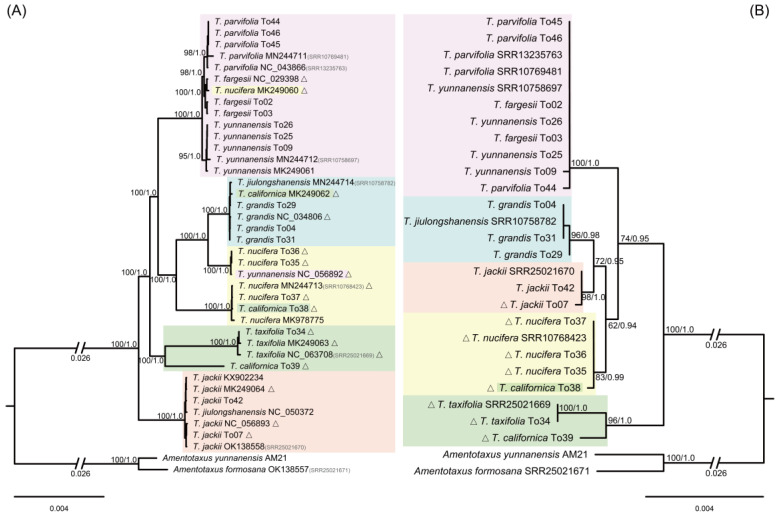
Phylogenetic relationships of *Torreya* species constructed using RAxML based on the filtered plastid (**A**) and nrDNA cistron (**B**) matrices. ML tree is shown with Maximum Likelihood bootstrap (BS)/Bayesian Inference posterior probability (PP) values given for each taxon node. Cultivated samples of *Torreya* are marked with triangles.

The unfiltered and filtered matrices of CP, nrDNA and MT yielded identical topologies, but the filtered matrices received relatively higher support values. Here, only the results from the filtered matrices (CP-gb, nrDNA-gb and MT-gb) are reported. The plastid phylogenetic tree showed that all taxon clades had high support values (BS ≥ 95%; PP = 1) (Figure 1A). Five out of the nine sampled species formed species-specific clades, except *T*. *nucifera*, *T*. *californica*, *T*. *jiulongshanensis*, and *T. yunnanensis*. The samples of *T*. *nucifera* formed a grade of two clades as sister to *T. grandis*. The three accessions of *T*. *californica* fell distantly in different clades, To39 as sister to the *T. taxifolia* clade, To38 in the larger *T. nucifera* clade, and MK249062 as sister to *T. jiulongshanensis* MN244714 in the *T. grandis* clade. The second *T. jiulongshanensis* sample, NC_050372, was placed in the *T. jackii* clade. One sample of *T*. *yunnanensis*, NC_056892, fell in the smaller *T. nucifera* clade (Figure 1A).

In the nrDNA-gb phylogenetic tree, five well to highly supported clades were received (BS ≥ 83%; PP ≥ 0.98) (Figure 1B), with four being species-specific, *T. grandis*, *T. jackii*, *T. nucifera*, and *T. taxifolia.* Additionally, here, the two *T*. *californica* samples fell in different clades, To38 in the *T. nucifera* clade, and To39 as sister to the *T*. *taxifolia* clade. Samples of three species, *T. fargesii*, *T. parvifolia*, and *T. yunnanensis*, fell in a polytomy. The single accession of *T*. *jiulongshanensis*, SRR10758782 (MN244714 in the plastome tree), fell into the clade of *T*. *grandis* (Figure 1B).

The mitochondrial phylogeny, MT-gb, showed low phylogenetic resolution (Figure S2). However, moderately to highly supported (BS ≥ 41%; PP ≥ 0.98) species-specific clades were found for *T. grandis*, *T. jackii*, *T. nucifera*, and *T. taxifolia*, as in the nrDNA-gb tree. The positions of the two *T*. *californica* samples were identical to those in the cpDNA-gb and nrDNA-gb trees. The single accession of *T*. *jiulongshanensis*, SRR10758782 (MN244714 in the plastome tree), fell into the *T. jackii* clade (Figure S2), different from its position in the plastome and nrDNA cistron trees where it was in the *T. grandis* clade (Figure 1). In the plastid tree, the other *T. jiulongshanensis* sample, NC_050372, was associated with the *T. jackii* clade. The phylogenetic relationships of the samples of *T*. *fargesii*, *T*. *parvifolia*, and *T*. *yunnanensis* were not well resolved (Figure S2).

**Figure 2:**
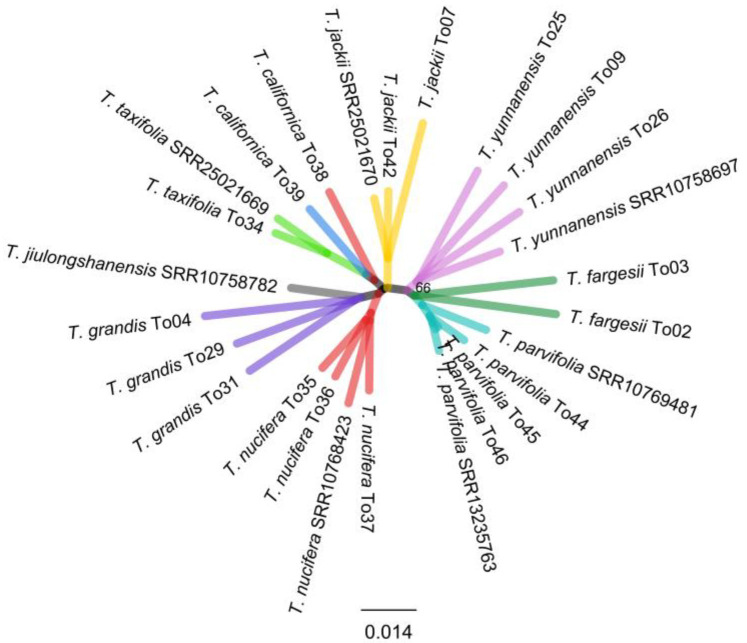
Skmer tree of *Torreya* species based on unassembled reads. Branches of each species are designated in different colors.

The Skmer analysis showed that all sampled *Torreya* species, except *T. californica*, with multiple accessions per species (i.e., excluding *T. jiulongshanensis* with one sample), were resolved in species-specific clades (Figure 2). These were basically the same grouping based on plastome and nrDNA cistron data (Figure 1). Although, the *T. californica* sample To38 was resolved with *T*. *californica* sample To39 and *T*. *taxifolia* clade, which was different in both the CP and nrDNA cistron trees.

### 2.3. Discordance between Plastid, nrDNA Cistron, and Mitochondrial Phylogenies

**Figure 3:**
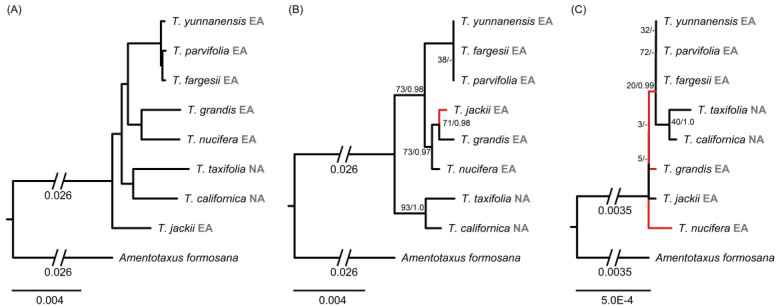
Phylogenetic relationships of *Torreya* species constructed using RAxML based on the filtered plastid (**A**), nrDNA cistron (**B**), and mitochondrial (**C**) matrices. ML tree is shown with Maximum Likelihood bootstrap (BS)/Bayesian Inference posterior probability (PP) values given for each node. Nodes without values represent maximal support in both ML and BI methods. Nodes with “-” represent unsupported relationship in the tree. Internal branches shown in red are not recovered in the left plastid tree. The species name is followed by distribution information, with NA for North America and EA for East Asia.

To compare phylogenetic species relationships inferred from different genomic data, one sample of each species that was correctly identified, based on next-generation DNA barcoding, was used for phylogenetic analysis. The interspecific relationships within *Torreya* were fully resolved with maximum support values using the complete plastome data (BS = 100%, PP = 1; Figure 3A). Here, *T. jackii* formed the earliest diverging species, and the clade of NA species, *T*. *taxifolia* and *T*. *californica*, was sister to the remaining species from East Asia. These formed two clades, i.e., *T. yunnanensis* (*T*. *parvifolia* + *T*. *fargesii*), and *T*. *nucifera* + *T*. *grandis*.

The nrDNA cistron phylogenetic tree showed similar species relationships compared to the plastome tree (Figure 3B). However, here *T*. *jackii* was resolved as sister of *T*. *grandis*, although not with high branch support all along the branches to the backbone (BS ≤ 73%; PP ≤ 0.98). The phylogenetic tree based on mitochondrial genes had low phylogenetic resolution and, in general, low branch support values (BS ≤ 72%; PP ≤ 1.0) (Figure 3C). *Torreya taxifolia* was sister to *T*. *californica* with low support value (BS = 40%), and *T*. *fargesii*, *T*. *yunnanensis* and *T*. *parvifolia* formed a polytomy (BS = 72%). Different from the phylogenies of plastome and nrDNA cistron sequences, the relationships between these two clades and the other three species, *T*. *grandis*, *T*. *jackii* and *T*. *nucifera*, were unresolved.

## 3. Discussion

### 3.1. Species Delimitation and Correction of Misidentified Accessions

The plastome was proposed as a super-barcode to distinguish species in Taxaceae with high species discrimination ability [11,12]. Recently, an alternative approach, Skmer, was proposed for sample identification, using unassembled genome skims, which can effectively improve the phylogenetic signal and identification resolution [50,51]. In the present study, all the nine *Torreya* species recognized in past treatments with multiple samples were analyzed with the next-generation DNA barcode and Skmer. All the 21 wild collected samples of the EA species formed species-specific clades, except *T. jiulongshanensis*, whose two sampled individuals fell into the *T*. *jackii* and the *T*. *grandis* clades, respectively (Figure 1), and was regarded a natural hybrid between *T*. *jackii* and *T*. *grandis* based on morphology and molecular markers [29] (see Section 3.2. below).

For the 17 accessions from botanical gardens/arboretums, 12 accessions grouped into their corresponding species clades and the one accession of *T. californica* (To39) formed a distinct lineage sister to *T. taxifolia*, while the remaining four accessions fell in other species clades in the plastome tree, indicating possible misidentification of these samples. Of these four, one accession (NC_056892) of ‘*T. yunnanensis*’ fell into the clade of *T*. *nucifera*, but not in the clade of *T. yunnanensis* including five accessions from wild populations. This accession was from an introduced plant in the Kunming Botanic Garden of the Chinese Academy of Sciences, which was confirmed to represent *T*. *nucifera* after checking this living collection. The same was the case for accession MK249060 of ‘*T*. *nucifera*’ from Lushan Botanic Garden (Jiangxi, China), which fell in the clade of *T*. *fargesii*, and was confirmed to represent a misidentification by examining a photo of the voucher for this accession [14]. Of the other two accessions (MK249062 and To38) identified as *T*. *californica* that fell in the *T*. *grandis* and *T*. *nucifera* clades, respectively, in the plastome tree, accession MK249062, used in Zhang et al. [30], was regarded misidentified and identified as *T*. *grandis* based on the result in our study (Figure 1 and Figure S1) as well as in Zhou et al. [14]. The accession To38, which was also sampled and sequenced in Zhou et al. [14] as Xianglab209, however, was not included for analysis in Zhou et al. [14], due to a high amount of missing data. In the present study, our resampled and sequenced accession To38/Xianglab209 had no missing data and fell in the clade of *T*. *nucifera* in the plastome, nrDNA cistron and MT trees, but it was resolved as a sister to clade of *T*. *californica* (To39) and *T*. *taxifolia* in the Skmer analysis. This indicates that it may be misidentified and, likely, its hybrid origin is between *T*. *nucifera* and *T*. *californica*. Therefore, accession To39 with a consistent placement in the phylogenies is assumed to represent a *T*. *californica* accession.

The accessions of *T*. *nucifera* formed two clades with high support in the plastome tree, indicating a possible cryptic species. However, the accessions of this species (To35, To36, To37, and SRR10768423) were grouped into a clade in the nrDNA cistron tree without any intraspecific genetic variation (Figure 1B). *Torreya nucifera* is endemic to Japan and South Korea, and the plastome is inherited maternally through seed. Therefore, plastid genome capture from *T*. *grandis* during an ancient secondary contact might be more feasible than a new cryptic lineage derived from *T*. *nucifera* for the clade sister to *T*. *grandis* in the plastome tree. Further detailed molecular and morphological studies are needed to elucidate this issue.

If the misidentified accessions are corrected and disregarding the putative hybrid *T. jiulongshanensis* and smaller *T*. *nucifera* clade, then all eight recognized species in *Torreya* sampled here were resolved as “good” species and were successfully discriminated based on the super-barcode of the complete plastid genome, as well as the Skmer analysis (excluding To38) with unassembled nuclear reads. It was notable that three species, *T*. *yunnanensis*, *T*. *fargesii* and *T*. *parvifolia*, were closely related and formed a single clade in nrDNA cistron and mitochondrial phylogenies without much variation (Figure 3), simply due to a shortage of variable sites, perhaps due to their short divergent time. Their relatively short branch lengths in the plastome phylogeny may further support their possibly recent allopatric divergence. However, the plastome tree and Skmer analyses allows the unambiguous delineation. Thus, it may be more appropriate to treat them at a subspecies level because they have distinct distribution range and a similar morphology, with only minor differences in seed traits [18,24]. Historically, Kang and Tang [52] treated *T. yunnanensis* as a variety of *T*. *fargesii*, and Farjon [18] accepted this taxonomic treatment. While Farjon [18] merged *T*. *parvifolia* into *T*. *grandis*, this was not supported in our study. On the other hand, Yi et al. [53] identified *T*. *parvifolia* as closely related to *T*. *yunnanensis*, and treated it as an independent species. Based on our results, we support the treatment of both *T. yunnanensis* and *T*. *parvifolia* as two varieties of *T*. *fargesii*, as *T*. *fargesii* var. *yunnanensis* and *T*. *fargesii* var. *parvifolia*, respectively.

Collectively, the proportion of misidentification of cultivated plants of *Torreya* held in botanical gardens was relatively high, being 23.5% (4 out of 17 accessions). Since the four accessions represented three species, the misidentification at species level was much higher with 37.5% (3 out of 8). In Zhang et al. [30], two of the seven sampled species were misidentified, which directly affected their inference of phylogenetic relationships and biogeographic history. In addition, species misidentification may also lead to mistakes in conservation-linked reintroduction programs, which reduces the scientific value of botanical garden/arboretum collections. Therefore, more caution should be taken when using cultivated plants from botanic gardens/arboretums for biological study, and there is a need for the verification of their species identification. In previous molecular studies [30,34,35], sequences were downloaded from GenBank or obtained from cultivated plants directly without concerns of species identification, which may have affected their phylogenetic inferences. The use of multiple samples per species in this respect cannot be overemphasized as only this allowed an independent check for correct species identifications [11]. At present, species identification of cultivated plants is of little concern, but fortunately some projects aim at comprehensive sequencing were conducted, such as sequencing for an entire botanical garden [54], which may provide a large amount of fundamental data for assessing identification issues of cultivated plants.

### 3.2. Hybrid Origin of T. jiulongshanensis

In this study, the non-monophyly of *T. jiulongshanensis*, already mentioned above, involved two sampled accessions falling into two clades representing *T*. *grandis* and *T*. *jackii* in the complete plastome tree (Figure 1). *Torreya jiulongshanensis* is endemic to South Zhejiang of East China and occurs within the sympatric region of *T*. *jackii* and *T*. *grandis*. Its morphological characters, especially leaf length and seed size, are intermediate between these two species [29]. Based on nuclear internal transcribed spacer (ITS) and plastid *rbcL* and *rpl16* sequences, Kou et al. [29] suggested *T*. *jiulongshanensis* to be a natural hybrid between *T*. *grandis* and *T*. *jackii* with the latter being the maternal parent due to the shared plastid haplotype with *T*. *jiulongshanensis*. It needs to be noted that the cytoplasm, i.e., plastome and mitochondria, in *Torreya* are apparently maternally inherited [41]. Given this, the fact that the two *T*. *jiulongshanensis* accessions fell with both species strongly suggests repeated and reciprocal hybridizations occurred. An alternative explanation may be leakage of the male cytoplasm, as observed for angiosperms and gymnosperms such as *Pinus* L. [37,55]. This might also explain the placement of one accession of *T. jiulongshanensis* (SRR10758782, the same accession of MN244714 in the plastome tree) with *T*. *grandis* in the plastome, nrDNA cistron trees, and Skmer analysis (Figure 1 and Figure 2), and grouping with *T*. *jackii* in the mitochondrial phylogeny (Figure S2). Overall, we can conclude that *T*. *jiulongshanensis* is a natural hybrid, in fact, more precisely, nothospecies *T*. *× jiulongshanensis*, between *T*. *jackii* and *T*. *grandis* with repeated bidirectional hybridization. To elucidate the exact cytoplasm inheritance patterns in *Torreya* would require further detailed studies.

### 3.3. Phylogenetic Relationships and Conflicts among the Three Genomic Phylogenies

Hybridization/introgressions, polyploidizations, and incomplete lineage sorting (ILS) may contribute to phylogenetic conflicts between nuclear and organelle phylogenies [36]. Discordances of the interspecific relationships within *Torreya* were revealed among the different genomic data. This was especially the case between the plastid topology on the one hand and the nuclear and mitochondrial topologies on the other (Figure 3). The unresolved relationship between the two species, *T. grandis* and *T. nucifera*, in the mitochondrial phylogenetic tree, as opposed to a sister relationship in the plastome tree, is likely an artefact of the low diversity of the MT sequences as shown in the low branch support. This is also reflected in the monophyly of the two NA species, *T*. *californica* and *T*. *taxifolia*, in the mitochondrial phylogenetic tree with the branch support of only 40%, compared with 93% and 100% in the nrDNA cistron and plastome trees, respectively. The main conflict between the phylogenies was in the placement of *T*. *jackii* as the first diverging lineage in the plastid tree, and as a member in a clade with *T. grandis* and *T. nucifera* in the nrDNA cistron tree. This was also found in previous studies [14,29,32]. Zhou et al. [14] suggested that the conflict was difficult to explain and that it could be the result of differential lineage sorting of a dimorphic ancestral chloroplast genome among species and clades of *Torreya*, or as the result of chloroplast capture by *T*. *jackii* from *Amentotaxus.* The latter, however, is very unlikely since it would require a recent hybridization event between very distant lineages. A more likely scenario is an introgressive hybridization event between *T*. *jackii* and *T*. *grandis*, with *T*. *jackii* being the maternal parent.

## 4. Materials and Methods

### 4.1. Sample Collection, DNA Extraction and Genome Skimming Sequencing

Our samples included 38 accessions of *Torreya* with multiple samples (2 to 6) of nine species following the most recent taxonomic classifications [18,20,28] (Table 1) that were sampled across their distribution ranges (Figure S1). For 19 of these, fresh leaves of either cultivated or wild field-collected individuals were sampled and dried immediately in silica-gel for DNA extraction. Voucher specimens were deposited at the Herbarium of Kunming Institute of Botany (KUN), Chinese Academy of Sciences. For the other 19 samples, the complete plastomes were downloaded from GenBank as available on 20 February 2023. The corresponding raw sequencing data (only available for seven accessions) were downloaded from the NCBI Sequence Read Archive (SRA) for nrDNA and mitochondrial genome assembly. Of these 38 samples, 21 came from the field and 17 came from arboretums/botanical gardens. *Amentotaxus yunnanensis* H.L. Li and *A*. *formosana* H.L. Li were selected as outgroups for data analyses (Table 1).

Total genomic DNA was extracted from silica-gel dried leaves using a modified CTAB method [56], and was quantified and sheared to a mean insert size of 500 bp for Illumina library construction with a TruSeq DNA Sample Prep Kit following the manufacturer’s instructions (NEBNext^®^ Ultra IITMDNA Library Prep Kit for Illumina^®^). The libraries were sequenced for each sample on an Illumina HiSeq X Ten platform (Illumina, San Diego, CA, USA) with 150 bp paired-end reads at BGI Wuhan, China. Adaptors and low-quality reads were filtered using fastp v0.21.0 [57] with default parameters. Improvement in read quality was checked using FastQC v0.11.5 (available from http://www.bioinformatics.babraham.ac.uk/projects/fastqc (accessed on 25 June 2018)).

### 4.2. Assembly and Annotation

The plastomes of the 19 newly sampled individuals were assembled from clean reads using the GetOrganelle toolkit [58]. The mitochondrial genomes and nrDNA cistrons of these newly sequenced individuals and the SRA data file reads were also assembled by GetOrganelle. In this pipeline, target-associated reads were recruited by Bowtie2 v2.3.4 [59], extracted from total genomic reads, and subsequently de novo assembled by SPAdes v3.15 [60]. Then, the slimmed assembly graph (FASTG) of each sample was visualized by Bandage [61] and the complete plastome and nrDNA cistron sequences were exported. The extracted plastome sequences of all newly sampled individuals, collineating with the reference (*Torreya grandis* NC_034806), were retained for subsequent analysis. Plastid genes were annotated using PGA [62] with the published plastome of *T. grandis* (NC_034806) as the reference and manually adjusted in Geneious v9.0.2 [63]. Transfer RNAs (tRNAs) were confirmed by tRNAscan-SE v2.0.3 [64]. The nrDNA cistrons were annotated using Geneious with *Chamaecyparis formosensis* (LC518080) as the reference. To obtain mitochondrial genes, the newly assembled mitochondrial genome scaffolds were annotated with the mitochondrial genome of *Taxus cuspidata* (MN593023) as the reference. The mitochondrial genes were then extracted from the annotated scaffolds using Geneious.

### 4.3. Phylogenetic Analyses

The complete plastome, mitochondrial genes and nrDNA cistron sequences of all accessions were aligned separately using MAFFT v7.407 [65], and manually adjusted where necessary in Geneious. The aligned sequences of the individual mitochondrial genes were concatenated in AMAS [66]. In order to assess the effect of alignment quality on the phylogeny, hypervariable and poorly alignable regions were filtered out in Gblocks v0.91b [67] using default parameters but with half-gap positions allowed.

We conducted phylogenetic analyses based on complete matrices and filtered matrices separately of the plastome (CP and CP-gb, respectively), mitochondrial genes (MT and MT-gb, respectively) and nrDNA cistrons (nrDNA and nrDNA-gb, respectively) with all accessions for species delineation using Maximum Likelihood (ML) and Bayesian Inference (BI) analyses. Before phylogenetic analysis of each matrix, PartitionFinder2 [68] was used to determine the best-fitting partitioning schemes and best-fitting nucleotide substitution models for each partition under the corrected Akaike information criterion (AICc). Linked-branch lengths with greedy search [69] was used for the matrices of mitochondrial genes and nrDNA cistrons and rcluster search [70] for the matrices of the whole plastome sequences. Each gene, intergenic region or intron (if any), was regarded as a predefined data block.

The best-fitting partitioning scheme and evolutionary model estimated for each subset were used for ML and BI analyses. The ML analyses were conducted using RAxML v8.2.12 [71] with the option of rapid bootstrap of 1000 replicates. The BI analyses were conducted using MrBayes v3.2.7a [72] with two independent runs each with four Markov chains. MrBayes was run for 10 million generations for the matrices of mitochondrial genes and nrDNA cistrons and 50 million generations for the matrices of the whole plastome, sampling every 1000 and 2500 generations, respectively. The first 25% of sampled trees were discarded as burn-in, and the remaining trees were used for generating the majority-rule consensus tree. The average standard deviation of split frequencies was ensured to reach a value less than 0.01, and the convergence of the MCMC chains was checked in Tracer v1.7.2 [73].

To determine the phylogenetic relationships within *Torreya* and obtain a species level tree, only one accession collected from the field, as far as possible, and correctly identified here for each species, excluding the hybrid *Torrey jiulongshanense*, was retained for these phylogenetic analyses. The partitioning scheme and nucleotide substitution model estimation, and ML and BI analyses were implemented as described above.

### 4.4. Skmer Analysis

In plant genome skimming data, most sequence data is from the nuclear genome. However, only ribosomal DNA sequences of the nuclear genome from the data are often used for species discrimination and plant phylogenetic studies, discarding a vast proportion of nuclear reads and only providing limited sequence information [44].

Skmer is an assembly-free method for estimating genomic distances between a query and reference genome skims, which can reflect the evolutionary divergence between two species for species identification [43]. We used Skmer v3.2.1 to analyze the unassembled nuclear reads of genome skimming data for *Torreya* species identification. Before Skmer analysis, reads of the organelle genome and the nrDNA cistron in the genome skimming data were mapped to their respective references constructed using mitochondrial genome of *Taxus cuspidate* mentioned above (MN593023), and plastid genome and nrDNA cistron sequences obtained in the present study, by Bowtie2, left unmatched reads (--un-conc-gz) as an input to Skmer. We used the workflow suggested by the authors to obtain the distance matrix for sequencing reads. Then, FastME v2.1.6.4 [74] was used to infer the backbone tree, and RAxML was used to generate the phylogenetic trees with support values.

## 5. Conclusions

We gathered wild and cultivated samples of *Torreya* with multiple accessions per species to evaluate a next-generation DNA barcode and Skmer for species delineation and sample identification, and to reconstruct interspecific phylogenetic relationships. All but one of the eight recognized species in *Torreya* were successfully discriminated based on super-barcode of the complete plastid genome (except *T. nucifera*) and the Skmer analysis (except *T*. *californica*), and *T*. *jiulongshanensis* was confirmed as a hybrid. The Skmer method uses unassembled nuclear reads from genome skims and significantly increased the discrimination rate compared to the nrDNA cistron and MT sequences, and could be proposed as a credible approach for species discrimination. We found that around a quarter of cultivated plants of *Torreya* were misidentified in botanical gardens/arboretums. This stresses the need for more caution when using cultivated plants and the need for careful verification of their identities. The interspecific phylogenetic relationships in *Torreya* were well-resolved with maximum support in the plastome phylogeny. Based on the robust phylogenetic topology, we preliminarily delineate the taxa greatly along recognized taxa from past treatments, with the exception of recognizing two varieties/subspecies in *T. fargesii*. This study enriches the existing genetic data of *Torreya*, and will provide genetic baseline data to facilitate the accurate species identification in the future and to confirm the identities of *Torreya* species in collections. The findings here shed light on the significance of the accurate species identification for biological studies, and also provide quantitative insights into the usage of cultivated samples for phylogenetic study.

## Figures and Tables

**Table 1:** Taxa and samples of *Torreya* and *Amentotaxus* included in the present study with information on voucher, locality, origin, and GenBank (Sequence Read Archive, SRA) accession numbers and NGS performance, and data sources.

Taxon	Correct Scientific Name	Voucher	Sample ID	GenBank (SRA) Accession Number of Plastome	GenBank (SRA) Accession Number of nrDNA Cistron	Locality Information	Origin	No. of Bases	No. of Reads	Plastome Size (bp)	No. of Reads Mapped to Plastome	Mean Coverage of Plastome (×)	Platome GC Content (%)	Source
*Amentotaxus formosana* *		-	AM10	OK138557 (SRR25021671)	(SRR25021671)	China: Taiwan, Pingtung		3,662,110,500	24,414,070	136,361	450,086	500	35.8	Wang et al. [11]
*Amentotaxus yunnanensis*		GBOWS079	AM21	OR197346	OR195109	China: Yunnan, Malipo, Xiajinchang		1,927,772,700	12,851,818	137,609	134,071	146.2	35.8	this study
*Torreya californica*	*Torreya nucifera*	Xianglab209	To38	OR197360	OR195123	USA: North Carolina JC Arboretum	cultivated	1,980,844,200	13,205,628	137,215	593,920	645.8	35.4	this study
*Torreya californica*	*Torreya grandis*	-	-	MK249062	-	UK: Edingburgh, Royal Botanic Garden Edinburgh	cultivated			136,693			35.4	Zhang et al. [30]
*Torreya californica* *		Xianglab276	To39	OR197361	OR195124	USA: Washington Arboretum	cultivated	3,243,790,200	21,625,268	136,957	429,684	468.5	35.4	this study
*Torreya fargesii*		Zhdq-195	To03	OR197348	OR195111	China: Gansu, Wenxian, Bikou	wild	4,098,586,800	27,323,912	137,055	261,206	279.2	35.5	this study
*Torreya fargesii*		-	-	NC_029398	-	China: Hubei, Wuhang, Wuhan Botanic Garden	cultivated			137,075			35.5	Tao et al. [45]
*Torreya fargesii* *		Zhdq-081	To02	OR197347	OR195110	China: Sichuan, Maoxian, Fengyi	wild	2,744,998,800	18,299,992	137,047	206,326	223.3	35.5	this study
*Torreya grandis*		LJ-10759	To31	OR197355	OR195118	China: Zhejiang, Linan, Tianmushan Nature Reserve	wild	3,982,402,800	26,549,352	136,991	487,543	556.7	35.4	this study
*Torreya grandis*		061013-8	To04	OR197349	OR195112	China: Jiangxi, Qianshan, Wuyishan Natural Reserve	wild	2,342,818,500	15,618,790	136,962	219,046	236.1	35.4	this study
*Torreya grandis*		-	-	NC_034806	-	China: Zhejiang, Hangzhou, Hangzhou Botanical Garden	cultivated			136,949			35.4	Miu et al. [46]
*Torreya grandis* *		ZLN-2011120	To29	OR197354	OR195117	China: Fujian, Nanping, Wuyishan Natural Reserve	wild	4,690,482,300	31,269,882	136,948	281,539	302.7	35.4	this study
*Torreya jackii*		GLM-07317	To07	OR197350	OR195113	China: Yunnan, Kunming, Kunming Botanical Garden	cultivated	2,766,930,600	18,446,204	136,798	398,596	433.6	35.5	this study
*Torreya jackii*		-	To43	OK138558 (SRR25021670)	(SRR25021670)	China: Jiangxi, Zixi, Matuoshan	wild	3,865,107,900	25,767,386	136,884	451,825	495	35.5	Wang et al. [11]
*Torreya jackii*		-	-	NC_056893	-	China: Yunnan, Kunming, Kunming Botanical Garden	cultivated			136,924			35.5	Ji et al. [31]
*Torreya jackii*		-	-	MK249064	-	China: Zhejiang, Hangzhou, Hangzhou Botanical Garden	cultivated			136,728			35.5	Zhang et al. [30]
*Torreya jackii*		-	-	KX902234	-	China: Zhejiang, Tonglu, Baiyunyuan Forest Park	wild			136,720			35.5	Li et al. [47]
*Torreya jackii* *		PVHJX03232	To42	OR197362	OR195125	China: Jiangxi, Zixi, Matuoshan	wild	3,073,291,500	20,488,610	136,751	343,308	374.3	35.5	this study
*Torreya jiulongshanensis*		-	-	NC_050372	-	China: Zhejiang, Jingning, Xikengxia Village	wild			136,705			35.5	Jiang et al. [48]
*Torreya jiulongshanensis*		-	-	MN244714 (SRR10758782)	(SRR10758782)	China: Zhejiang, Suichang, Jiulong Mountain Nature Reserve	wild	3,842,990,166	25,450,266	137,320	574,469	755.8	35.4	Miao et al. [35]
*Torreya nucifera*		19940566	To35	OR197357	OR195120	USA: Atlanda, Atlanta Botanical Garden	cultivated	6,329,296,224	42,242,036	136,944	630,323	685.3	35.5	this study
*Torreya nucifera*		19940647	To36	OR197358	OR195121	USA: Atlanda, Atlanta Botanical Garden	cultivated	5,910,584,366	39,445,718	136,944	1,051,344	1144.5	35.5	this study
*Torreya nucifera*		-	-	MK978775	-	South Korea: Pyungdae-ri, Jeju-do Island	wild			136,985			35.4	Shin et al. [49]
*Torreya nucifera*	*Torreya fargesii*	-	-	MK249060	-	China: Jiangxi, Jiujiang, Lushan Botanical Garden	cultivated			136,970			35.5	Zhang et al. [30]
*Torreya nucifera*		-	-	MN244713 (SRR10768423)	(SRR10768423)	China: Jiangshu, Nanjing, Nanjing University	cultivated	3,495,500,208	23,149,008	136,955	238,714	261.5	35.5	Miao et al. [35]
*Torreya nucifera* *		19980776	To37	OR197359	OR195122	USA: Atlanda, Atlanta Botanical Garden	cultivated	5,231,130,270	34,919,542	137,276	247,792	269.2	35.4	this study
*Torreya parvifolia*		5025	To44	OR197363	OR195126	China: Sichuan, Tuowu, Wuyi Town	wild	7,353,802,222	49,770,264	137,160	694,509	779.7	35.5	this study
*Torreya parvifolia*		W24	To46	OR197365	OR195128	China: Sichuan, Tuowu, Wuyi Town	wild	7,520,255,400	50,135,036	137,183	587,446	639	35.5	this study
*Torreya parvifolia*		-	-	NC_043866 (SRR13235763)	(SRR13235763)	China: Sichuan, Liangshan	wild	12,258,497,819	81,276,138	137,106	512,642	561.7	35.5	Zhang et al. [34]
*Torreya parvifolia*		-	-	MN244711 (SRR10769481)	(SRR10769481)	China: Sichuan, Butuo, Wuyi, Wandun Mountain	wild	4,853,642,830	32,143,330	136,781	264,197	290.5	35.5	Miao et al. [35]
*Torreya parvifolia* *		W21	To45	OR197364	OR195127	China: Sichuan, Tuowu, Wuyi Town	wild	7,492,564,200	49,950,428	137,198	342,748	374.4	35.5	this study
*Torreya taxifolia*		-	To33	NC_063708 (SRR25021669)	(SRR25021669)	USA: Atlanda, Atlanta Botanical Garden	cultivated	5,860,851,364	39,120,638	137,117	452,291	498.8	35.4	Wang et al. [11]
*Torreya taxifolia*		-	-	MK249063	-	USA: Atlanda, Atlanta Botanical Garden	cultivated			137,285			35.4	Zhang et al. [30]
*Torreya taxifolia* *		20121421	To34	OR197356	OR195119	USA: Atlanda, Atlanta Botanical Garden	cultivated	5,707,656,428	38,089,352	136,972	444,007	485.5	35.4	this study
*Torreya yunnanensis*		GLM-07342	To09	OR197351	OR195114	China: Yunnan, Yulong, Xinzhu	wild	3,523,821,900	23,492,146	137,074	506,145	549.1	35.5	this study
*Torreya yunnanensis*		GLM-092567-1	To26	OR197353	OR195116	China: Yunnan, Weixi, Weideng	wild	4,241,168,700	28,274,458	137,122	399,311	430.7	35.5	this study
*Torreya yunnanensis*	*Torreya nucifera*	-	-	NC_056892	-	China: Yunnan, Kunming, Kunming Botanical Garden	cultivated			137,412			35.4	Ji et al. [31]
*Torreya yunnanensis*		GLM-07342-1	-	MK249061	-	China: Yunnan, Yulong	wild			136,844			35.5	Zhang et al. [30]
*Torreya yunnanensis*		-	-	MN244712 (SRR10758697)	(SRR10758697)	China: Yunnan, Weixi	wild	4,151,493,132	27,493,332	136,807	233,613	256	35.5	Miao et al. [35]
*Torreya yunnanensis* *		GLM-07469	To25	OR197352	OR195115	China: Yunnan, Gongshan, Bingzhongluo	wild	4,142,196,600	27,614,644	137,029	280,765	301.4	35.5	this study

* The sample used for phylogenetic analysis at species level.

**Table 2:** Comparison of characteristics in the alignments of unfiltered and filtered (-gb) matrices of the plastid genome (CP), the 26 isolated mitochondrial genes (MT), and the nuclear ribosomal DNA cistron (nrDNA) across *Torreya* samples.

Dataset	No. of Samples	Length (bp)	Length (bp) *	Variable Sites (%) *	PI Sites (%) *	Identical Sites (%) *
CP	40	145,775	140,787	2561 (1.82%)	2063 (1.47%)	138,226 (98.18%)
CP-gb	40	135,852	135,852	2258 (1.66%)	1860 (1.37%)	133,594 (98.34%)
MT	27	21,493	21,385	14 (0.07%)	7 (0.03%)	21,371 (99.93%)
MT-gb	27	21,303	21,303	13 (0.06%)	6 (0.03%)	21,290 (99.94%)
nrDNA	28	6137	6115	52 (0.85%)	44 (0.72%)	6063 (99.15%)
nrDNA-gb	28	6100	6100	52 (0.85%)	44 (0.72%)	6048 (99.15%)

* The information of aligned length, variable sites, parsimony-information (PI) sites, and identical sites of *Torreya* (excluding two samples of *Amentotaxus*).

## Data Availability

Sequence alignments and sequences of plastome, nrDNA cistron and mitochondrial gene have been deposited in Science Data Bank at: https://doi.org/10.57760/sciencedb.09201.

## Supplementary Materials

The following supporting information can be downloaded at: https://www.mdpi.com/article/10.3390/ijms241713216/s1.
