# Supplementary material for: Phylogenetic Relationships and Next-Generation Barcodes in the Genus Torreya Reveal a High Proportion of Misidentified Cultivated Plants
Source: Int J Mol Sci. 2023 Aug 25;24(17):13216. doi: 10.3390/ijms241713216 (PMC10487542; doi:10.3390/ijms241713216)
Supplement: Supplementary file 1 [file ijms-24-13216-s001.zip › FigureS1 sample_distribution.pdf]

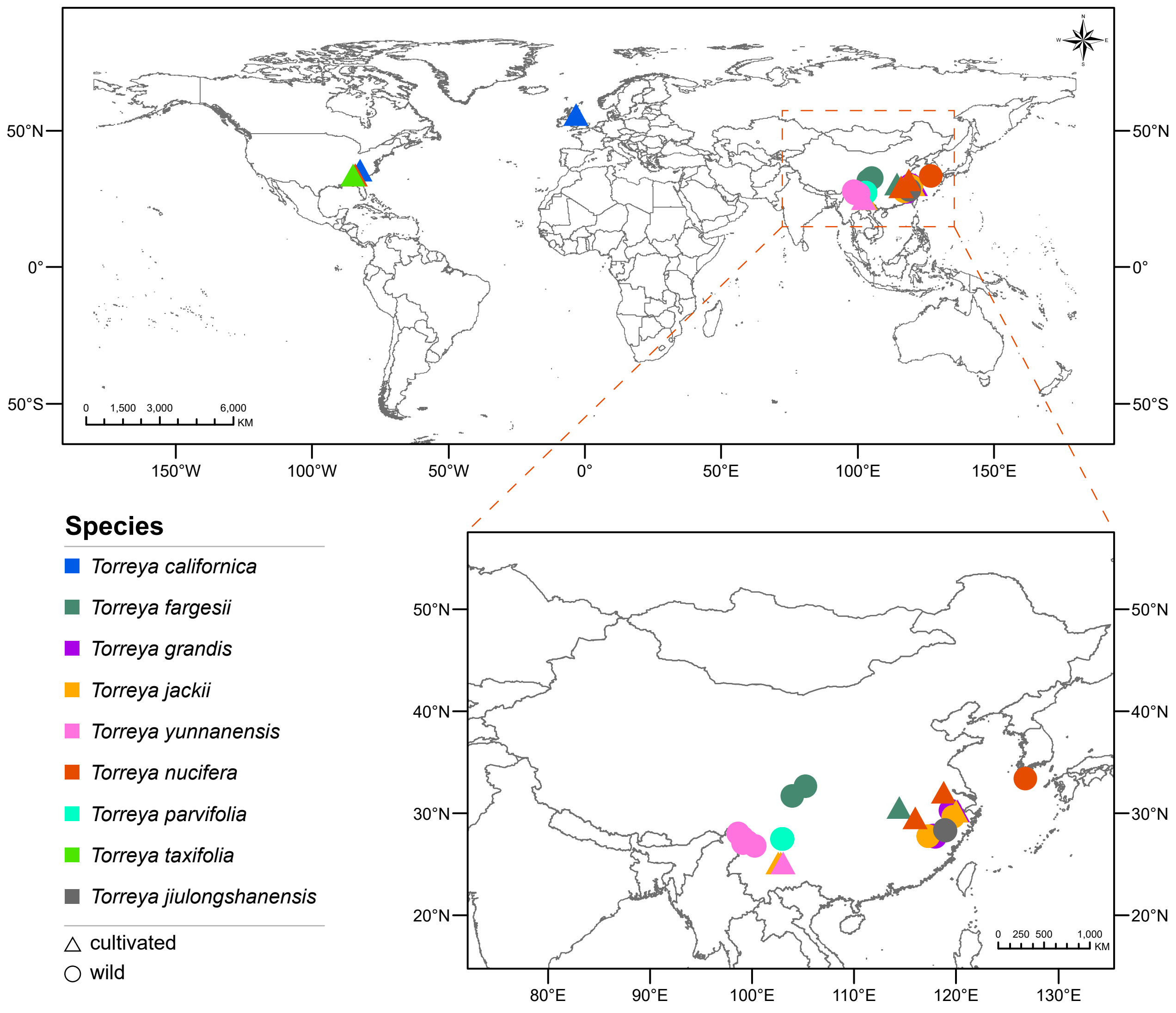

Figure S1. Sampling locations of *Torreya* species, each of which is designated with a different color. A triangle and a circle indicate the cultivated and wild source of each sample, respectively.
