# Supplementary material for: Phylogenetic Relationships and Next-Generation Barcodes in the Genus Torreya Reveal a High Proportion of Misidentified Cultivated Plants
Source: Int J Mol Sci. 2023 Aug 25;24(17):13216. doi: 10.3390/ijms241713216 (PMC10487542; doi:10.3390/ijms241713216)
Supplement: Supplementary file 1 [file ijms-24-13216-s001.zip › FigureS2 27-mt-gb.ML-BI.tre.pdf]

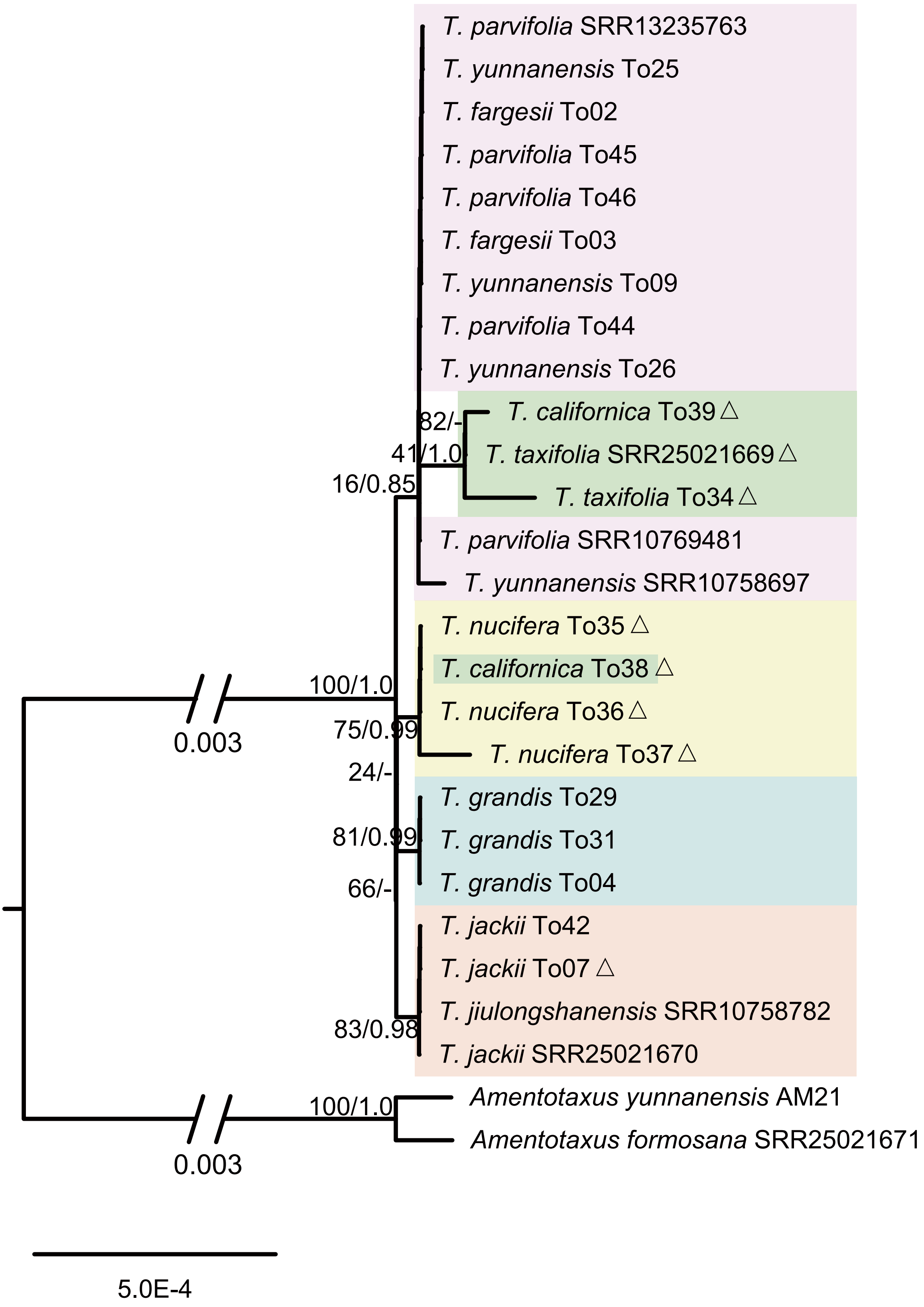

Figure S2. Phylogenetic relationships of *Torreya* constructed using RAxML based on the filtered mitochondrial matrix. ML tree is shown with Maximum Likelihood bootstrap (BS)/ Bayesian Inference posterior probability (PP) values given for each taxon node. Cultivated samples of *Torreya* are marked with triangles.
